# Adaptive Mechanisms of Tumor Therapy Resistance Driven by Tumor Microenvironment

**DOI:** 10.3389/fcell.2021.641469

**Published:** 2021-03-01

**Authors:** Peijie Wu, Wei Gao, Miao Su, Edouard C. Nice, Wenhui Zhang, Jie Lin, Na Xie

**Affiliations:** ^1^School of Basic Medical Sciences, Chengdu University of Traditional Chinese Medicine, Chengdu, China; ^2^State Key Laboratory of Biotherapy and Cancer Center, West China Hospital, and West China School of Basic Medical Sciences & Forensic Medicine, Sichuan University, and Collaborative Innovation Center for Biotherapy, Chengdu, China; ^3^Department of Biochemistry and Molecular Biology, Monash University, Clayton, VIC, Australia; ^4^Department of Medical Oncology, The Second Affiliated Hospital of Kunming Medical University, Kunming, China

**Keywords:** therapeutic resistance, tumor microenvironment, adaptive resistance, exosome, immunotherapy, cancer-associated fibroblasts, vasculature system, hypoxia

## Abstract

Cancer is a disease which frequently has a poor prognosis. Although multiple therapeutic strategies have been developed for various cancers, including chemotherapy, radiotherapy, and immunotherapy, resistance to these treatments frequently impedes the clinical outcomes. Besides the active resistance driven by genetic and epigenetic alterations in tumor cells, the tumor microenvironment (TME) has also been reported to be a crucial regulator in tumorigenesis, progression, and resistance. Here, we propose that the adaptive mechanisms of tumor resistance are closely connected with the TME rather than depending on non-cell-autonomous changes in response to clinical treatment. Although the comprehensive understanding of adaptive mechanisms driven by the TME need further investigation to fully elucidate the mechanisms of tumor therapeutic resistance, many clinical treatments targeting the TME have been successful. In this review, we report on recent advances concerning the molecular events and important factors involved in the TME, particularly focusing on the contributions of the TME to adaptive resistance, and provide insights into potential therapeutic methods or translational medicine targeting the TME to overcome resistance to therapy in clinical treatment.

## Introduction

Cancer is a significant public health problem worldwide, with substantial incidence and mortality rates (Ferlay et al., 2019). There have been spectacular advances in the development and therapeutic application of treatment for tumors, including chemotherapy, radiotherapy, targeted therapy, and immunotherapy over the past several decades (Szakács et al., 2006). However, resistance to these therapies has been a major obstacle that restricts the effectiveness of cancer treatments and impacts patient survival (Liu et al., 2018). Therefore, most patients respond to therapies at an early stage, whereas patients at a later stage frequently display poor clinical outcomes with continuous treatment (Miller et al., 2019). A broad range of intrinsic mechanisms underlying how cancer cells escape from the cytotoxicity of tumor therapies have been revealed, including decreased drug accumulation, altered drug metabolism, mutated or altered drug target and enhanced DNA repair capability, as well as inactivated cell death signaling (Assaraf et al., 2019; Milman et al., 2019; Valencia and Kadoch, 2019; Zhong and Virshup, 2020). Tumor cell heterogeneity, especially cancer stem-like cells (CSCs), is another cause leading to various resistance responses for multiple therapies (Housman et al., 2014; Steinbichler et al., 2018). Accordingly, multiple studies have addressed intracellular response, including genetic or epigenetic alterations, for cell survival under the death pressure induced by therapies (Cheng et al., 2020; Jiang W. et al., 2020; Long et al., 2020; Wang et al., 2020). New viewpoints and theories have proposed that tumor progression, especially when confronted with external pressure from various therapies, is a dynamic and complicated process that tightly interacts with the surrounding environment (Hanahan, 2014).

The tumor microenvironment (TME) is the extracellular environment in which tumors cells exist, and consists of carcinogenetic cells, cancer-associated fibroblast (CAFs), immune cells [including T and B lymphocytes, tumor-associated macrophages (TAMs), and natural killer cells], the vasculature system, and the extracellular matrix (ECM; including secreted cytokine, chemokine, metabolites, and exosomes) (Quail and Joyce, 2013; Belli et al., 2018). It has been shown that the non-malignant cells in the TME are not just silent bystanders, but rather actively boost carcinogenesis by promoting excessive tumor initiation, malignant progression, metastasis, and therapeutic resistance (Mantovani et al., 2008; Grivennikov et al., 2010; Hanahan and Weinberg, 2011; Balkwill et al., 2012; Hanahan and Coussens, 2012). The transformed cancer cells are found to interact with stromal cells in the TME, which contribute extensively to tumor development and resistance. Additionally, hyperplasia, metabolic remodeling, malignant proliferation, and inhibition of apoptosis in tumor cells contribute to hypoxia, oxidative stress, and acidosis within the TME. These abnormal conditions further modulate the ECM to induce angiogenesis or mechanical stiffness, and ultimately result in metastasis and resistance (Schrader et al., 2011; Quail and Joyce, 2013; Maman and Witz, 2018; Lin et al., 2019). CAFs induce cancer progression as well as therapeutic resistance through the secretion of cytokines or chemokines, exosomes, and ECM remodeling factors (Shiga et al., 2015; Fu et al., 2016). Macrophages, adipocytes, and fibroblasts in the TME can also act as a sanctuary for tumor cells to escape immune elimination (Hui and Chen, 2015). Therefore, combination chemotherapy with drugs targeting the TME, such as immune cells and angiogenesis, has had success in clinical trials for overcoming drug resistance (Correia and Bissell, 2012; Jo et al., 2018).

**FIGURE 1:**
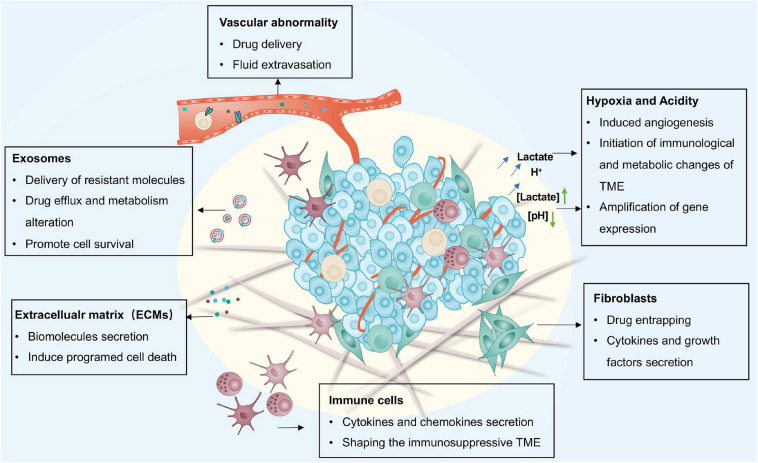
The main adaptive mechanisms driven by the TME for therapy resistance including CAFs, immune cells, vasculature system, ECM, exosomes, hypoxia, and acidity. CAFs, cancer-associated fibroblasts; ECM, extracellular matrix.

Accordingly, an interesting concept has been proposed that the resistance of tumor cells to multiple therapies may be caused by both active (cell-autonomous) and adaptive (non-cell-autonomous) mechanisms. While numerous active mechanisms of therapeutic resistance have been summarized previously, here, we focus on the adaptive resistance to various therapies mainly dependent on the TME (Figure 1) (Holohan et al., 2013; Housman et al., 2014). Furthermore, the complex reciprocity between tumors and TME and its role in adaptive resistance is discussed, with a perspective on prospects of overcoming therapeutic resistance.

## TME-Driven Adaptive Mechanisms of Therapy Resistance

The TME contains a wide variety of cell types including CAFs, immune cells, and vascular cells embedded in the ECM. The TME also contains exosomes, metabolites and cytokines that mediate heterocellular interactions. Moreover, the physical or chemical features, including hypoxia, acidity, and oxidative stress, all facilitate tumor progression and resistance.

### Cancer-Associated Fibroblasts (CAFs)

**FIGURE 2:**
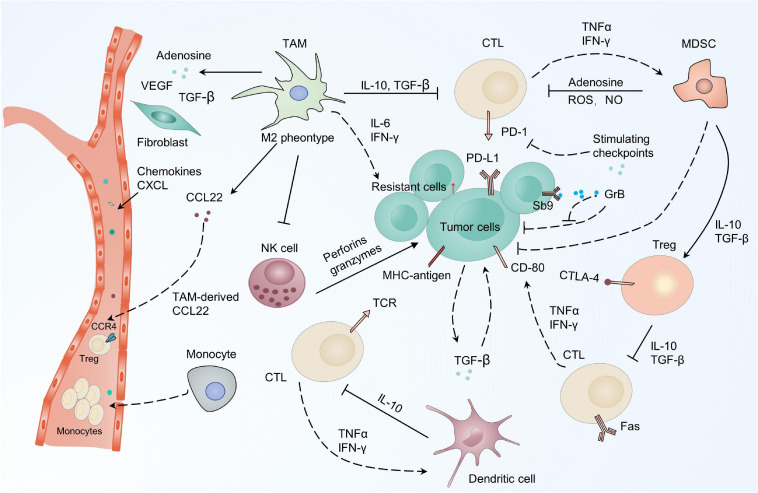
Immune system response for resistance in the TME. Scavenging of tumor cells by the immune system is mainly mediated by CTLs which can be inhibited by cytokines and chemokines secreted by several types of immune cells in the TME. There are also many molecules on the tumor surface which contribute to exhausting, or even eliminating, anti-tumor immune cells like CTLs. TME, tumor micro-environment; CTLs, cytotoxic T cells.

Cancer-associated fibroblasts are a prevalent subpopulation of cells in the tumor stroma (Orimo et al., 2005; Räsänen and Vaheri, 2010). The conversion from quiescence to activation of fibroblasts provokes various oncogenic signals that facilitate tumor cells to escape therapies (Kalluri and Zeisberg, 2006). Co-culture of prostate tumor cells with CAFs attenuates doxorubicin cytotoxicity by obstructing DNA damage and suppressing ROS generation in tumor cells (Cheteh et al., 2017). CAFs may also be responsible for the resistance of therapy through secreting chemokines, growth factors, metabolites, and exosomes, causing resistance and recurrence (Figure 2) (Billottet et al., 2008; Kojima et al., 2010; Räsänen and Vaheri, 2010; Straussman et al., 2012). CAF-secreted PAI-1 activates the AKT and MAPK pathways in a paracrine way to reduce chemotherapy drug-induced DNA damage, ROS generation, and cell death in esophageal squamous cell carcinoma (ESCC) (Che et al., 2018). HGF secreted by CAFs can combine with the MET receptor to activate the PI3K-Akt and MAPK pathways, which is responsible for the resistance of BRAF inhibitors or EGFR inhibitors to glioblastoma, colon cancer, and melanoma (Thomasset et al., 1998; Luraghi et al., 2014; Fiori et al., 2019). In addition, CAF-derived paracrine signals including chemoattractant cytokines, metabolites, and exosomes induce the NF-κB pathway, contributing to tumor cell resistance (Sun et al., 2012; Chan et al., 2016; Su et al., 2018; Zhang D. et al., 2018). Treatment with chemotherapeutic drugs upregulates WNT16B in CAFs, which mitigates the cytotoxic effects of drugs through the NF-κB pathway in prostate cancer cells (Sun et al., 2012). Similarly, secreted IL-1β and the constitutively expressed IL-1 receptor associated kinase 4 (IRAK4) induce the activation of the NF-κB pathway both in CAFs and pancreatic cancer cells, alleviating the cytotoxicity of gemcitabine in pancreatic tumors (Zhang D. et al., 2018). IL-6 is another cytokine released by CAFs that promotes cisplatin resistance through the STAT3/NF-κB pathway by upregulating CXCR7 in ESCC (Qiao et al., 2018).

Given the pivotal role of CSCs in therapeutic resistance, the CAFs strengthen stemness as a route of acquired resistance (Zhao, 2016; Fiori et al., 2017). The IL-6 and IL-8 released by CD10^+^GPR77^+^ CAFs can promote CSCs sustaining chemotherapy resistance in breast and lung tumors (Su et al., 2018). Meanwhile, chemotherapy-treated colorectal CAFs promote the self-renewal of CSCs by increasing the secretion of interleukin-17A (IL-17A) (Lotti et al., 2013). In addition, TGF-β2 secreted by CAFs cooperate with HIF-1α derived from the hypoxic TME to activate the Hedgehog pathway, which promotes cancer cell stemness and resistance to chemotherapy (Tang et al., 2018). Moreover, ELF chemokines secreted by CAFs are also proven to induce the transformation of tumor cells to stem cells in breast and pancreatic tumors (Chan et al., 2016). The secretion of FGF5 by Hedgehog-activated CAFs in mouse models of breast cancer creates a supportive microenvironment for cancer cells by fostering a reversible stem-like phenotype. Indeed, inhibition of Hedgehog signaling by Smo inhibitors can hinder the transformation toward stemness status to recover the sensitivity of cancer cells to docetaxel (Cazet et al., 2018).

Furthermore, the complicated crosstalk between CAFs and tumor cells also contributes to resistance. For example, epithelial expression of platelet-derived growth factor (PDGF)-CC causes the CAFs to secrete STC1, IGFBP3, and HGF, which are responsible for the tamoxifen resistance in breast cancer (Roswall et al., 2018). To escape tumor treatment, the upregulated insulin receptor (IR) and insulin-like growth factor (IGF) 1 receptor (IGF1R) in cholangiocarcinoma (CCA) cancer cells promote the proliferation and activation of CAFs. CAF-secreted IGF2 provides a feedback pathway with IR/IGF1R to induce the resistance of cancer cells to erlotinib, a tyrosine kinase inhibitor (TKI). Hence, an IR/IGF1R inhibitor can improve the deleterious effect of erlotinib in xenografts models (Vaquero et al., 2018). Additionally, cancer cell-derived serum components, such as lysophosphatidic acid (LPA) and proteases, are reported to stimulate CAFs remodeling for tumor cells survival (Park et al., 2008; Yarnold and Brotons, 2010; Mantoni et al., 2011; Calvo et al., 2013). The remodeled CAFs, however, exert paracrine actions on tumor resistance via secreted growth factors, including VEGF-A, TGF-β, and various cytokines. CAFs-tumor cell contact can also activate the NOTCH signaling pathway, facilitating stroma-mediated radiotherapy (Freund et al., 2010; Acosta et al., 2013).

In general, the above evidence has shed light on the adaptive mechanism that CAFs utilize with tumor cells for acquired resistance in many cancers including lung, breast, prostate, and glioblastoma. CAFs can interact with various TME factors including immune cells, tumor cells, and the ECM to participate in tumor cell resistance. The heterogeneous nature of CAFs and their multiple functions are interesting potential research directions as they offer a promising strategy for novel cutting-edge therapies directed at tumors and the TME.

### Immune Cells

**FIGURE 3:**
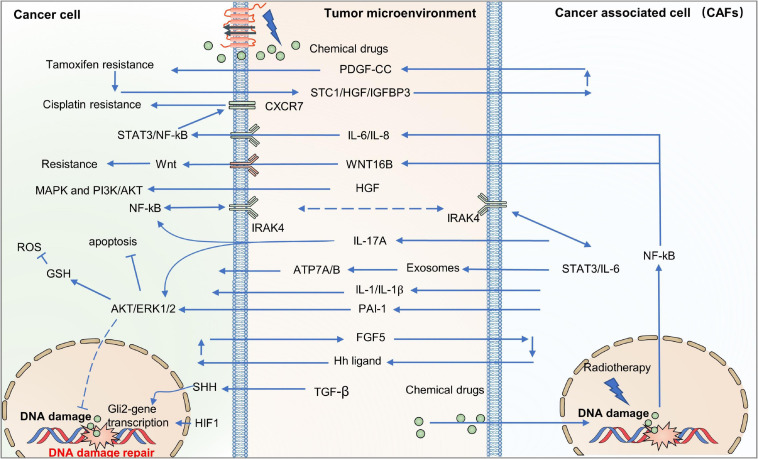
CAFs drive resistance-related paracrine pathways in the TME. CAFs provide an adaptive response for resistance by secreting chemokines, growth factors, metabolites, and exosomes, which activate various signaling pathways in cancer cells, including PI3K-Akt/MAPK, NF-κB, and STAT3. PI3K-Akt, the phosphatidylinositol 3-kinase (PI3K)/protein kinase B (AKT) signaling pathway; MAPK, mitogen-activated protein kinase pathway; NF-κB, nuclear factor-κB; STAT3, signal transducer and activator of transcription 3.

The immune cells in the TME mainly consist of B cells, effector and regulatory T cells, TAMs, myeloid-derived suppressor cells (MDSCs), natural killer cells (NKs) as well as dendritic cells (DCs) (Chen F. et al., 2015; Lei et al., 2020). These cells are crucial for tumorigenesis, exerting either promoting or antagonizing effects on tumors (Lei et al., 2020). Cytotoxic CD8^+^ T cells (CTLs) are the primary lymphocytes for killing tumor cells. They secrete cytotoxic enzymes including perforin and granzyme, and can interact with the major histocompatibility complex I (MHC-I) (Tanaka et al., 1999; Jackaman et al., 2012; Martínez-Lostao et al., 2015; Wei et al., 2018; Zhong et al., 2020). The involvement of CTLs in tumor therapeutic resistance is proven by the strong association between the profile of CTLs in tumors and the chemotherapy outcomes (Figure 3) (Denkert et al., 2010; Halama et al., 2011; Chen and Chang, 2019). In ovarian cancer, CTLs can enhance the immunogenic action of IFN-β to abrogate CAF-mediated resistance to platinum-based chemotherapy (Wang et al., 2016). Indeed, the combination of cisplatin with immune checkpoint inhibitors (ICIs) have been shown to result in better clinical outcomes (Gandhi et al., 2018; Socinski et al., 2018). TAMs are one of the most abundant cells detected in solid tumors and are derived from circulating monocytes. They also play an important role in controlling the immunosuppressive mechanisms of the TME, contributing to tumor development and therapeutic resistance (Solinas et al., 2010; Ruffell and Coussens, 2015; Räihä and Puolakkainen, 2018; Salmaninejad et al., 2019; Saleh and Elkord, 2020). Evidence implicates TAMs in the secretion of cytokines including IL-6 and IFN in response to resistance (Jinushi et al., 2011; Salvagno et al., 2019). Blocking colony stimulating factor 1 receptor (CSF-1R) signaling to regulate the polarization status of TAMs has been found to be an effective way of restoring the sensitivity of cisplatin involving IFN response (Salvagno et al., 2019). Accordingly, these promising results have encouraged the evaluation of combination therapy strategies targeting the immune system for tumor patients.

Growing evidence suggests that immunotherapy has shown dramatic efficacy in clinical outcomes (Wei et al., 2018; Cervantes-Villagrana et al., 2020). However, some cancer patients treated using this approach show limited response rates due to acquired resistance (O’Donnell et al., 2017; Sharma et al., 2017). It has been shown that acquired immune resistance may be achieved by three approaches: (i) increased levels of immunosuppressive cells and molecules; (ii) upregulation of immune checkpoints; and (iii) tumor mutation loads and loss of target antigens. For example, enhanced recruitment of immunosuppressive cells, such as TAMs and MDSCs, reduces the sensitivity of immunotherapy and enhances immunosuppression, leading to acquired resistance (Restifo et al., 2016; O’Donnell et al., 2017; Sharma et al., 2017). Maj et al. (2017) have demonstrated that apoptotic Tregs can upregulate the level of extracellular adenosine which can be correlated with acquired resistance to anti-PD-L1 mAb treatment in mice models. Moreover, the compensatory inhibitory mechanism also contributes to acquired resistance. For instance, increased CD4^+^TIM-3^+^ and CD8^+^TIM-3^+^ T cells in lung tumor biopsies has been related with resistance to anti-PD-1 mAb in both humans and mice (Koyama et al., 2016). Similarly, increased TIM-3 on CTLs has been reported in HNSCC following treatment with anti-PD-1 mAb (Shayan et al., 2017). The alteration of neoantigens may be another reason for acquired resistance, as manifested by the fact that loss of CD19 causes resistance following CD19-CAR-T cell therapy (Shalabi et al., 2018). Interestingly, the latest studies implicate that the serine protease inhibitor SerpinB9 (Sb9), expressed in CAFs, MDSCs, and TAMs, can promote tumor cell proliferation by combining with granzyme B (GrB) leading to resistance to immunotherapy (Mangan et al., 2017; Jiang L. et al., 2020; Yin et al., 2020).

### Vasculature System

Numerous studies have shown that the responsiveness of tumors to therapy is affected by the vasculature system. Mechanically, the vasculature influences drugs transportation and sensitivity by controlling the supply of nutrients and oxygen, ultimately regulating tumor cell survival (Galmarini et al., 2000). The vasculature transports nutrients and oxygen, as well as other growth factors, to both normal and tumor tissue, and removes waste products following cellular metabolism, which can be involved in tumor relapse, metastasis, and resistance. Vessels in tumors are observed to be convoluted, branched, and dilated, with excessive loops compared with normal tissues (Jain, 1988). In some cases, vessels cannot be transformed into capillaries, arterioles, or venules. Moreover, vessel walls are often discontinuous or absent compared with normal tissues leading to varying degrees of leakiness in different tumors (Benjamin et al., 1999; Carmeliet and Jain, 2000; Hashizume et al., 2000; Yonenaga et al., 2005; Trédan et al., 2007). Accordingly, blood flow in tumors becomes chaotic and variable (Carmeliet and Jain, 2000). In the tumor vascular network, the viscosity and geometric resistance of blood is increased, and the pressure between arterioles and venules is depressed (Sevick and Jain, 1989; Trédan et al., 2007). Further, in normal tissues, pressure is reduced via the lymphatic network. However, lymph vessels are found to be lacking or have reduced functionality in solid tumors compared with normal tissues, leading to higher interstitial pressure (Leu et al., 2000; Stohrer et al., 2000; Heldin et al., 2004; Trédan et al., 2007). This higher interstitial pressure can suppress the transportation and distribution of larger biological molecules, and diverts blood away from the center toward the periphery of the tumor (Salnikov et al., 2003; Heldin et al., 2004). Interestingly, even in the same tumor, the rate of blood flow or morphology of vessels may vary with space and time (Gillies et al., 1999; Vaupel, 2004). These aberrant vasculature systems, and the dimensional compression of vessels caused by the excessive proliferation of tumor cells, reduce the rate of blood flow and impair the nutrition and oxygen supply to the tumor tissues (Padera et al., 2004). The insufficient supply of nutrients and oxygen as well as clearance of metabolic waste creates an acidic and hypoxic TME, which promotes therapeutic resistance (Tatum, 2006).

Further, the synergistic effect of various cell types in the TME, such as pericytes, endothelial cells, and bone marrow-derived progenitors, is the basis of tumor angiogenesis, which is reported to be sensitive to oxygen levels (Weis and Cheresh, 2011; Semenza, 2013). Mesenchymal stem cells (MSCs), TAMs, and CAFs all promote tumor angiogenesis by releasing various angiogenesis-related ligands. For instance, increased VEGFA is associated with poor prognosis in metastatic tumors such as lung, colon, and renal cell carcinomas (Hegde et al., 2013; Lee and Wu, 2015).

The delivery of drugs is also compromised by these aberrant vasculature systems (Durand, 2001). The infiltration gradient in the spatial distance from vascular components to tumor lesions is related to the distribution of drugs from the tissues to cancer cells. Microvascular density (MVD) is an important index for the clinical outcomes of carcinomas of the lung, breast, and liver (Trédan et al., 2007; Ariotti et al., 2015; Yuan et al., 2015). Growing evidence indicates that VEGF receptor inhibitor resistance is mainly caused by proangiogenic factors, suggesting that combination with anti-angiogenic agents may improve clinical outcomes compared with VEGF receptor inhibitors alone (Flaherty et al., 2015). In addition, the chemokine CXC motif ligand receptor (CXCR7) is reported to promote angiogenesis by increasing ERK1/2 phosphorylation (Yamada et al., 2015). Interestingly, the CXCL12-CXCR7 complex has been shown to mediate pro-angiogenesis, tumor growth, lung metastasis, and resistance (Yamada et al., 2015; Sun, 2016). Accordingly, anti-angiogenesis may be an affective therapy by specifically targeting tumor blood vessels.

### Extracellular Matrix (ECM)

The ECM consists of fibrous protein (such as laminin, elastin, and collagen), proteoglycans, water, and microelements, weaving a complex fiber-based network to provide structural support and regulate cellular activities, including proliferation, communication, and adhesion (Watnick, 2012; Martino et al., 2014; McAndrews et al., 2015; Korneev et al., 2017; Walker et al., 2018). In tumors, both the composition and physical or chemical properties of the ECM are different, depending on the tumor tissue, resident cells, tumor staging, and therapeutic strategies (Shree et al., 2011; Correia and Bissell, 2012; Watnick, 2012; Klemm and Joyce, 2015; Sato et al., 2016; Korneev et al., 2017; Jo et al., 2018; Senthebane et al., 2018; Walker et al., 2018). The ECM contributes to tumor resistance by influencing drug delivery, facilitating the escape from immune surveillance, and manipulating the transmembrane signaling transduction process.

Drugs are usually transported to the tumor issues by the pressure of blood circulation through interstitial areas. In this process, drugs need to cross the physical and biochemical barriers of the TME. The desmoplastic stroma has been found to be a barrier responsible for drug resistance by impeding the delivery of anti-cancer drugs and affecting vascular systems in tumors (Olive et al., 2009). In interstitial spaces, the organization of the ECM has been found to increase fluid pressure due to the barriers of the tumor mass, significantly suppressing the efficacy of drug delivery (Correia and Bissell, 2012; Maeda and Khatami, 2018). Moreover, excessive proliferation of cancer cells promotes fluid flux from the neoplasms toward the surroundings which impedes drug transportation (Chen Y. et al., 2019). Indeed, drug delivery efficiency has been demonstrated to be inhibited in the 3D cultured spheroids compared with the 2D monolayer owing to the density of the ECM cells (Jo et al., 2018). Furthermore, tumor cells within the collagen I matrix display obvious resistance when cells are exposed to 5-fluorouracil/oxaliplatin (Kanazawa et al., 2017; Jo et al., 2018; Matsunuma et al., 2019).

Besides influencing drug delivery, the ECM also plays an essential role in controlling cytokine activity, of which TGF-β is the most important. TGF-β induces the recruitment of fibroblasts to the tumor site and transformation to CAFs by regulating ECM matrix degradation (Itoh et al., 2017; Paauwe et al., 2018; Purcell et al., 2018). In addition, TGF-β along with HIF-1 can induce lysyl oxidase (LOX) which orchestrates ECM stiffness by inducing cross-linked collagen (Dauer et al., 2018; Zhao et al., 2018). ECM stiffness, in turn, can activate the TGF-β signaling pathway to form a bridge in the basement membrane and contribute to tumor cell evasion (Upagupta et al., 2018; Najafi et al., 2019). Indeed, genomic and transcriptomic analysis have demonstrated that the activated gene sets in response to TGF-β signaling are involved in regulating various pathophysiological processes including EMT, wound healing, angiogenesis, and dissemination (Hugo et al., 2016). On the other hand, TGF-β can regulate immune response by orchestrating the crosstalk of multiple cell types in the TME, including CAFs, lymphocytes, and endothelial cells (Korneev et al., 2017; Chakravarthy et al., 2018; Kesh et al., 2020). TGF-β can inhibit the proliferation and differentiation of anti-tumor T cells by increasing the expression of CD25 and Foxp3 (Löffek, 2018). Moreover, TGF-β induces the secretion of monocyte chemoattractant protein-1 (MCP-1) to upregulate the expression of mesenchymal markers and chemotactic factors (CCL-2, 7, 8, 13), which are associated with anti-PD-1 immune resistance (Díaz-Valdés et al., 2011; Sawa-Wejksza and Kandefer-Szerszeń, 2018).

Among the multiple TME factors that impact cancer cell therapy resistance, cell adhesion to the ECM has been considered as a key determinant (Eke and Cordes, 2015). In particular, cell adhesion-mediated drug resistance depends on interactions between integrins and ECM components such as collagen, fibronectin, and laminin (Grivennikov et al., 2010; Hanahan and Weinberg, 2011; Korneev et al., 2017; Arneth, 2019; Cassim and Pouyssegur, 2019). Integrin-mediated resistance has been reported to influence chemical drugs, radiotherapy, and targeted therapies such as TKIs (Goel et al., 2013; Seguin et al., 2014). It has been reported that the treatment of fibronectin or collagen-deficient ECMs with cisplatin increases the sensitivity of tumor cells by about 40% (Senthebane et al., 2018). The loss of integrin subunits, such as αvβ3 or αvβ5, can significantly restore the sensitization of glioblastoma and breast tumor cells to radiotherapy (Cao et al., 2006; Belli et al., 2018). The silencing of the αv subunit also increases the efficacy of oxaliplatin in colon tumor cells (He et al., 2009; Cruz da Silva et al., 2019). Additionally, the over-expression of β1 integrin significantly inhibits cell death in hepatocellular carcinoma exposed to etoposide, cisplatin, or docetaxel (Zhang et al., 2002, 2019; Ogawa et al., 2008). Mechanically, integrins transmit the signals in the microenvironment into intracellular pathways through focal adhesion kinase (FAK) and integrin-linked kinase (ILK) (Eke and Cordes, 2015). The complexes of FAK, ILK, cortactin, and cysteine-histidine-rich 1 as well as parvin α have been reported to inhibit the outcome of radiotherapy (Eke and Cordes, 2015). Integrin β1 regulates the dephosphorylation of FAK to protect tumor cells resistant to radiotherapy in a JNK-dependent manner on HNSCC and PDAC *in vitro* and *in vivo* (Goel et al., 2013; DeRita et al., 2019). Blocking the α5β1 subunit reduces the resistance to ellipticine and temozolomide dependent on p53 mutation status in glioblastoma cells (Martinkova et al., 2010; Fujita et al., 2020). Upregulated α4 integrin is responsible for the resistance in AML and esophageal cancer cells via the PI3K/Akt pathway (Layani-Bazar et al., 2014; Chen and Chang, 2019; Cruz da Silva et al., 2019). Additionally, a few findings suggest that the NF-κB or ILK-RhoB pathways may be involved in integrin-mediated resistance (Monferran et al., 2008; Ahmed et al., 2013). The ECM proteoglycan, versican, can impact immune surveillance evasion along with hyaluronan by increasing the expression of inflammatory cytokines such as IL-6, TNFα, and NF-κB (Kang et al., 2017; Gordon-Weeks and Yuzhalin, 2020; Wight et al., 2020; Gamradt et al., 2021). Taken together, the complexity of the ECM in composition and structure as well as heterogeneity still needs to be further understood for therapeutic purposes.

### TME-Derived Exosomes

**FIGURE 4:**
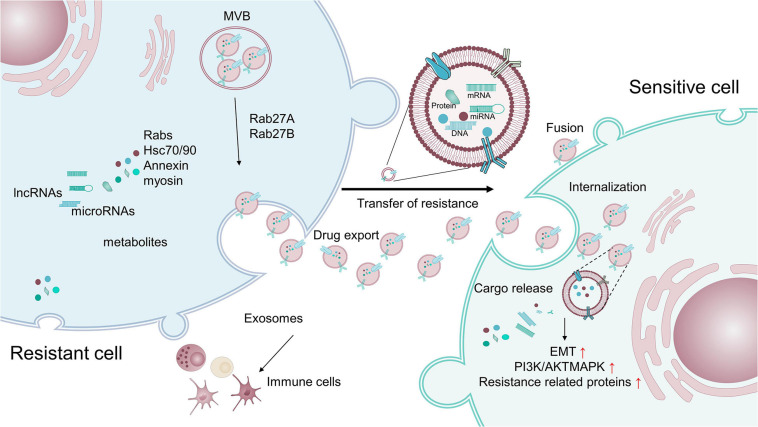
Exosomes transfer therapy resistance between resistant cells and sensitive cells. Exosomes containing cargo, such as proteins and non-coding RNAs, that are related to pro-survival, anti-apoptosis, and drug-efflux factors, can promote the acquisition of resistance in tumor cells by modulating various processes, including the reduction of intracellular drug concentrations, induction of EMT, activation of anti-apoptotic pathways, alteration of critical survival signal transduction pathways, and modulation of the immune system.

Exosomes or extracellular vesicles (EVs) with sizes of 40 to 100 nm, originating from large multivesicular bodies (MVBs), mediate cell-to-cell communication by transferring biologically active cargo, including DNAs, RNAs, proteins, and metabolites (Schneider and Simons, 2013; Sun, 2016; Mashouri et al., 2019). Exosomes have been demonstrated to be crucial signaling mediators in the TME, participating in tumorigenesis, metastasis, TME remodeling, angiogenesis, and therapeutic resistance (Figure 4) (Luga et al., 2012; Yoshizaki et al., 2013; Jeppesen et al., 2014; Sung et al., 2015; You et al., 2015; Paolillo and Schinelli, 2017; Wang X. et al., 2018; Zeng et al., 2018; Mashouri et al., 2019; Steinbichler et al., 2019; Yang E. et al., 2020). For example, exosomes have been found to control metabolic reprogramming (Yang E. et al., 2020). Exosomes can also scavenge unfavorable molecules in normal cells. Cancer cells hijack exosomes for the efflux of anti-cancer drugs, resulting in drug resistance (Safaei et al., 2005). Thus, drug-resistant ovarian carcinoma cells exhibit an enhanced exosomal export of cisplatin together with putative transporters MRP2, ATP7A, and ATP7B (Safaei et al., 2005).

Exosomes and their cargos can also promote the drug resistance of target cells (Shedden et al., 2003; Corrado et al., 2013). Exosomes extracted from resistant breast and prostate cancer cells have been shown to contain MDR1/P-gp transporters, conferring resistance to drug-sensitive tumor cells (Levchenko et al., 2005; Corcoran et al., 2012; Lv et al., 2014). miR-155 in exosomes has been reported to induce chemo-resistance by increasing the FOXO-3a, TGF-β, and C/EBP-β-mediated expression of EMT markers (Du and Shim, 2016; Crow et al., 2017; Lobb et al., 2017; Wang et al., 2019). Notably, exosomes isolated from triple-negative breast cancer cells can induce drug resistance on non-tumorigenic breast cells by modulating the PI3K/AKT, MAPK, and HIF1A pathways (Ozawa et al., 2018). Additionally, lymphoma exosomes carrying CD20 shield target cells form an antibody attack via the complement consumption of therapeutic anti-CD20 antibodies, leading to evasion of humoral immunotherapy (Aung et al., 2011).

Intriguingly, exosomes also promote therapeutic resistance by facilitating the intricate crosstalk between tumor cells and non-tumor cells within the TME. RNAs within exosomes derived from stromal cells activate the pattern recognition receptor RIG-I and downstream STAT1 signaling, which facilitates the growth of resistant tumor cells (Boelens et al., 2014). Exosomes secreted by TAMs transfer miR-21 to gastric cancer cells, which activates the PI3K/AKT signaling pathway and suppresses cell apoptosis to confer resistance (Zheng et al., 2017). Meanwhile, TAMs can directly deliver mRNAs to enhance the expression of CDK6, mTOR, STAT3, and β-catenin, leading to cisplatin resistance in BCa cells (Wu et al., 2020). In another example, exosomes released from CAFs strengthen the chemo-resistance of pancreatic cancer cells and colorectal cancer cells by activating β-catenin and the Snail pathway (Richards et al., 2017; Ren et al., 2018).

Collectively, exosomes can cause tumor cells to acquire resistance by various pathways, including the reduction of intracellular drug concentrations, induction of EMT, activation of anti-apoptotic pathways, alteration of critical survival signal transduction pathways, and modulation of the immune system (Mashouri et al., 2019; Steinbichler et al., 2019; Dai et al., 2020).

### Hypoxia, Acidity, and Oxidative Stress

The abnormal vasculature and heavy requirement for oxygen in tumors create a chronic or diffusion-limited hypoxia and acid environment (Bussink et al., 2003; Heldin et al., 2004; Milosevic et al., 2004; Gargiulo et al., 2019; Zaidi et al., 2019). The hypoxic environment of tumors leads to a decreased supply of nutrients such as glucose and essential amino acids (Lee et al., 2014; Tan et al., 2015; Ma et al., 2020). Moreover, tumor cells prefer to undergo glycolysis rather than oxidative metabolism which often converts glucose to lactate for the production of ATP, resulting in sequential acidic microenvironments (Dang and Semenza, 1999; Singh et al., 2017; Moloney and Cotter, 2018). Furthermore, a decreased capability of removing these acidic products results in low interstitial pH, another feature of solid tumors (Tran et al., 2016; Singh et al., 2017). These hypoxic and acidic conditions will induce aberrant activation of oncogenic pathways and genetic instability, contributing to tumor development and resistance (Li and Sun, 2018). For example, hypoxia in tumors increases the expression of multiple genes associated with angiogenesis and cell survival by activating HIF-1, a basic helix-loop-helix transcription factor (bHLH) (Pouysségur et al., 2006; Chen and Sang, 2016; Petrova et al., 2018; Lee et al., 2020). Serving as an oxygen sensor, HIF-1 may promote the expansion of tumor cell populations and alteration of biochemical metabolites involved in a resistant phenotype in response to hypoxia. It has been shown that hypoxia reduces sensitivity to p53-mediated apoptosis and promotes chemotherapeutic resistance in tumor cells (Kinoshita et al., 2001; Holle et al., 2016; Ritter, 2017). In solid tumors, the activation of major oncogenic signaling pathways such as Ras and PI3K/AKT, and the silencing of tumor suppressors LKB1, PTEN, and TSC2/1 can activate HIF-1, contributing to resistance (Shaw et al., 2004; Shackelford et al., 2009). Stabilization of HIF-1α through interaction with Hsp90/Hsp70 also facilitates cell survival under stress conditions (Luo et al., 2010; Taipale et al., 2010). Additionally, HIF-1α can cooperate with CAF-secreted TGF-β2 to induce GLI2 signaling cancer stem cells, leading to enhanced stemness and chemotherapy resistance (Tang et al., 2018).

Mechanically, HIF-1 activation induces the transcription of genes that facilitate pathophysiological alterations related to resistance, including the suppression of apoptosis and the induction of drug efflux and metabolism (Warfel and El-Deiry, 2014; Xia et al., 2018). Apoptosis may be a major factor in cell death induced by chemo- or radio- therapies (Krakstad and Chekenya, 2010; Mohammad et al., 2015; Zhao et al., 2015). Interestingly, HIF-1α has been found to both inhibit proapoptotic proteins, including TRAIL, and activate anti-apoptotic proteins, such as survivin, c-myc, STAT3, and TCF4, to promote the survival of tumor cells under chemo- or radio- therapies (Pei et al., 2010; Rohwer et al., 2010; Nishimoto et al., 2014; Zhao et al., 2016). HIF-1α also influences sensitivity to therapy through regulation of genes related to metabolic pathways (Lu et al., 2011; Meijer et al., 2012; Huang et al., 2013). HIF-1α can upregulate GLUT-1 to promote glycolysis, leading to increased intracellular ATP, pyruvate, and lactate levels (Meijer et al., 2012), while the suppression of HIF-1α results in a reduced glucose uptake, decreased lactate production, and increased oxygen species (ROS), which contribute to the enhanced efficacy of radiotherapy (Meijer et al., 2012). Hypoxia has been reported to promote temozolomide (TMZ) resistance in glioblastoma multiforme (GBM), through the activation of HIF-1α and NF-κB, followed by upregulated expression of Bcl-xL (Kitange et al., 2009; Chen W.L. et al., 2015). Transient hypoxia has also been found to cause an increase in dihydrofolate reductase and P-glycoprotein, which contributes to the resistance of drugs targeting topoisomerase II (Kovacic and Osuna, 2000; Gray et al., 2005). Interestingly, oxygen concentration may affect the efficacy of the anti-cancer drugs doxorubicin and mitomycin C, by delivering electrons to the oxygen (Brown and Wilson, 2004; Trédan et al., 2007; Yang G. et al., 2020).

Acidity in the TME has been demonstrated to affect the efficacy of various therapies. It can influence the transport of chemical drugs across the membrane due to the pH gradient caused by the acidic extracellular pH and near neutral or alkaline intracellular pH in tumors (Gerweck et al., 2006; Trédan et al., 2007). Hence, drugs with an acidic dissociation constant of 7.5–9.5 may show a significantly reduced rate of uptake, such as vincristine, mitoxantrone, doxorubicin, and vinblastine (Cowan and Tannock, 2001; Gerweck et al., 2006; Trédan et al., 2007; Zhou et al., 2019). Therefore, the cytotoxicity of these drugs is suppressed, resulting in a resistant phenotype (Raghunand et al., 2003; Trédan et al., 2007; Stepka et al., 2020). However, the concentration of some weakly acidic drugs including cyclophosphamide and chlorambucil may be increased in the neutral intracellular region (Gerweck et al., 2006; Trédan et al., 2007). Moreover, an acidic TME also alters the cellular proteome, cellular metabolism, and signaling pathways, facilitating stemness and drug resistance in cancer cells. The acidosis induces SOX2 expression by inhibiting vitamin D receptor (VDR)-mediated transcription, resulting in drug resistance (Hu et al., 2020). Enhanced lactate uptake and oxidation-induced lactic acidosis foster the resistance to uprosertib, a pan-Akt inhibitor, in colon cancer cells (Barnes et al., 2020). Moreover, the acidic environment induces the activity of p-glycoprotein (pGP) by activating p38 signaling, leading to multi-drug resistance in rat prostate cancer cells (AT1) (Sauvant et al., 2008). Clearly, the acidity of the TME must be considered when designing the delivery of drugs to obtain maximal therapeutic effect.

Oxidative stress is another feature of the TME, which is caused by the overproduction of reactive oxygen species (ROS) from both tumor cells and non-malignant cells in the TME. Oxidative stress plays a pivotal role in tumor progression, particularly through immune cell suppression. ROS downregulates the anti-tumor functions of effector immune cells that are recruited to the tumor site, notably T lymphocytes and natural killer (NK) cells which mediate anti-tumor immunity. MDSCs have been found to inhibit T cell proliferation to promote colorectal cancer cell proliferation by increasing ROS levels (OuYang et al., 2015; Weinberg et al., 2019). ROS and peroxynitrite in MDSC trigger the nitration of the TCR/CD8 complex which inhibits its interaction with pMHC, contributing to T cell tolerance and tumor escape (Nagaraj et al., 2007). ROS generated from NOX2-sufficient myeloid cells inhibits the NK cell-mediated clearance of malignant cells, facilitating the metastasis of melanoma cells (Aydin et al., 2017). The NK cells residing in the tumor core or primed by IL-15 exhibit higher thiol densities that can prevent other lymphocytes from ROS within the TME (Yang Y. et al., 2020). High levels of ROS following TCR and CD28 stimulation enhance Treg cell-mediated tumor immunosuppression and attenuate anti-tumor T cell responses by stabilizing SENP3 (Yu et al., 2018). In conclusion, oxidative stress acts as an important mediator of anti-tumor immunity. Achieving targeted oxidative stress could be a potential strategy to improve the efficacies of existing immunotherapy treatment.

### Heterocellular Metabolic Interactions

Growing evidence has demonstrated that disordered metabolism in the TME plays a crucial role in malignancy, metastasis, and immune resistance. The impact of metabolism on immune-resistance is mainly caused by two aspects: a reshaped immunosuppressive TME from tumor metabolic stress and immune-inhibiting metabolites generated by tumor cells.

Cancer cells usually exhibit high rates of glycolysis and aggressive depletion of amino acids such as tryptophan, arginine, and glutamine compared with normal cells. Tumor metabolic stress modulates the metabolic properties of malignant cells, which in turn influences nutrient shortage, oxygen competition, and acidity in the TME to create an immune-resistant environment (Martinez-Outschoorn et al., 2017). It is known that the demand for nutrients is especially high in TME, and this nutrient competition can impair the anti-cancer immune response. For example, the competition of carbohydrates can inhibit the anti-tumor effect of cytotoxic T cells by inducing the expression of immunosuppressive cytokines and immune checkpoint inhibitors (Wu et al., 2021). The restriction of glucose supplies due to the high rates of glycolysis in tumor cells impairs the anti-tumor function of CD4^+^ T cells, possibly by blocking the secretion of IFN-γ (Chang et al., 2015; Ho et al., 2015). Similarly, L-arginine deprivation has been found to inhibit anti-tumor immunity by inducing MDSC infiltration or suppressing the toxicity of IFN-γ. L-arginine depletion also promotes the immune evasion of cancer cells by elevating the tumoral level of PD-L1 (Morrow et al., 2013; Fletcher et al., 2015; Hugo et al., 2016; Prima et al., 2017; Kim et al., 2018; Jiang Z. et al., 2020). Tryptophan is another crucial amino acid that contributes to the anti-tumor immune response by regulating the kynurenine metabolic pathway (Jiang Z. et al., 2020; Xie et al., 2020). Indoleamine 2,3-dioxygenase (IDO), an essential enzyme of the tryptophan metabolic pathway, controls the production of kynurenine which exerts immunosuppressive effects by inducing differentiation of T cells and reduces immunogenicity (Wang et al., 2015; Ramapriyan et al., 2019). Both inhibitory immune cells such as MDSCs, DCs, and M2 macrophages as well as tumor cells can express IDO (Ramapriyan et al., 2019). Cancer cells have also been found to reduce tryptophan levels in TME to inhibit immune response (Ramapriyan et al., 2019). Besides glucose and amino acid metabolic pathways, lipid-related metabolism also plays an essential regulatory role in immunosuppressive function (Kamphorst et al., 2013). Obesity induces a desmoplastic TME by promoting inflammation and TAN infiltration, leading to impaired response to chemotherapy in PDAC. Reversal of obesity-aggravated desmoplasia by blocking the angiotensin-II type-1 receptor (AT1) improves response to chemotherapy. Meanwhile, cholesterol depletion can also recover the cytotoxic effect of chemical agents on PDAC and HCC (Guillaumond et al., 2015; Incio et al., 2016). Fatty acids have also been demonstrated to determine the cell fate of T cells and CD8^+^ effector T cells (O’Sullivan et al., 2014).

In addition, a variety of tumor metabolites have been reported to promote immune evasion. Glutamine is the most abundant amino acid in the TME, playing an essential role in anabolic growth and metastasis (Pusch et al., 2017; Zhang et al., 2017). Recent research has demonstrated that glutamine metabolism can impair anti-cancer immune response (Zhang et al., 2017; Oh et al., 2020). Glutamine blockade causes increased glucose and glutamine levels in the TME, inducing MDSCs apoptosis, promoting their differentiation toward the M1 type, and sensitizing resistant tumor cells to immunotherapies (Zhang et al., 2017; Leone et al., 2019; Oh et al., 2020). Adenosine, the breakdown production of ATP, can active the adenosine-AR pathway to escape from the killing effects of the immune system by reducing the response of NK cells, M1 macrophages, and CD8^+^ effector T cells. In addition, methylglyoxal (MG), a side-production of glycolysis, is an immunosuppressive metabolite that promotes tumor cell growth and resistance (Nokin et al., 2017; Antognelli and Talesa, 2018; Antognelli et al., 2019; Oh et al., 2020). The high concentration of methylglyoxal in the TME derived from MDSCs leads to the accumulation of methylglyoxal in T cells, contributing to anti-tumor evasion (Baumann et al., 2020). DMBG (*N-N*-dimethylbiguanide) can recover the sensitivity of immunotherapy-resistant tumor cells by removing the glycation activity of methylglyoxal (Baumann et al., 2020). Another molecule that can establish an immunosuppressive TME is lactate, a product of glycolysis and glycogenolysis (Gabrilovich et al., 2012; Mantovani et al., 2017; Pucino et al., 2017; Tong et al., 2018; García-Cañaveras et al., 2019). Lactate induces the development of MDSCs, polarization of macrophages into an immunosuppressive phenotype, maturation of DCs, and inhibition of effector T cells, thereby promoting immune evasion (Husain et al., 2013; Brand et al., 2016; Laoui et al., 2016; Angelin et al., 2017; Morrot et al., 2018). Furthermore, lactate can control CAFs to produce growth factors including hyaluronan (Wu et al., 2017; Apicella et al., 2018; Feichtinger and Lang, 2019).

Collectively, tumor cells reshape their metabolism adaptively which leads to the remodeling of the TME. The heterocellular metabolic interactions create an immunosuppressive TME, which subsequently enhances tumor proliferation and immune-therapy resistance. Thus, targeting tumor cell metabolism or metabolites in the TME should have great potential for recovering immunotherapy resistance.

### The Epithelial Pathway in Response to TME

**FIGURE 5:**
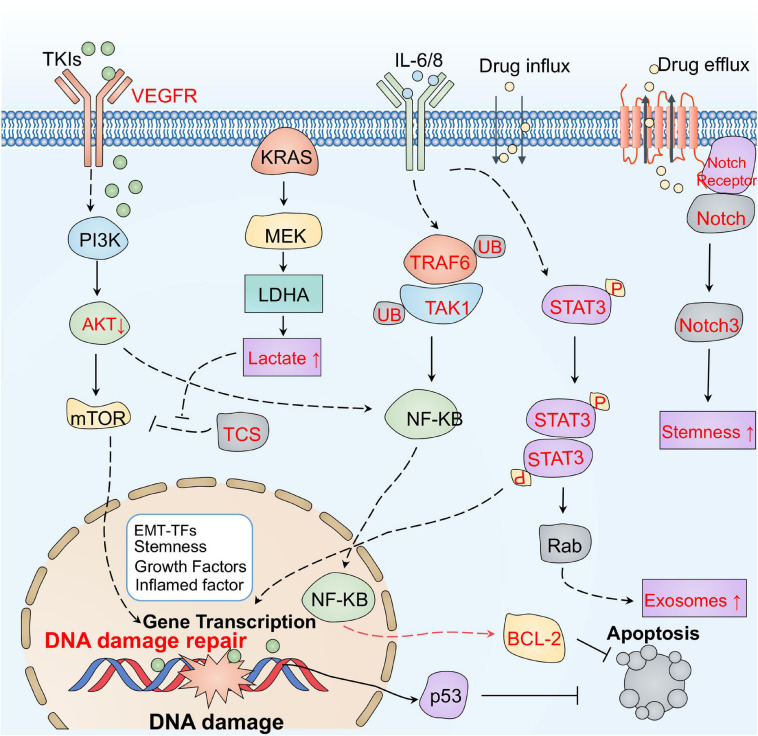
The main signaling pathways responsible for therapeutic resistance mediated by the tumor environment. Tumor cells can adaptively inhibit oncogenic AKT, which induces the secretion of inflammatory molecules such as IL-6/8, and EVs to restrict damage during therapy. The ATM-TRAF6-TAK1 axis related with DNA damage may also be involved in resistance. Some metabolites, such as lactate, in the TME have been reported to activate the mTOR pathway through glutamine metabolic pathways, inducing resistance to VEGF inhibitors. In addition, the STAT3 pathway may also rapidly respond to cytokines, promoting the secretion of exosomes, by upregulating Rab and increasing anti-apoptotic signaling in the TME. IL-6 and exosomes derived from the stroma deliver Jag1, Notch ligand, and Notch3 activate the Notch pathway to trigger resistance.

It is well known that tumor initiation and progression rely on bidirectional communications between tumor cells and the associated environment. Several signaling pathways in tumor cells, including Akt, mTOR, STAT3, and Notch, may be responsible for the altered tumor environment exposed to tumor therapies (Figure 5). Surprisingly, tumor cells can adaptively inhibit oncogenic AKT, which induces the secretion of inflammatory molecules such as IL-6/8 and extracellular vehicles (EVs) to restrict the damage induced by therapy (Salony et al., 2016). Thus, suppression of AKT signaling can increase drug resistance in tumor cells (Manning and Cantley, 2007). Moreover, mTOR acts as a vital protein to regulate cell growth both in physiological and pathological conditions (Guri and Hall, 2016). It has been shown that the secretome in the TME can activate mTOR signaling after treatment. Blocking mTOR signaling can restore the sensitivity of several anti-tumor drugs including crizotinib, vemurafenib, and erlotinib (Obenauf et al., 2015). Additionally, the ATM-TRAF6-TAK1 axis related with DNA damage may be involved in these processes (Zhang B. et al., 2018). Surprisingly, some metabolites in the TME, such as the lactate, have been reported to activate the mTOR pathway through glutamine metabolic pathways, inducing the resistance to VEGF inhibitors (Allen et al., 2016).

In addition, the STAT3 pathway can rapidly respond to cytokines, including L-1β and IL-6 released from neutrophils, TAMs, and CAFs in the TME (Samavati et al., 2009; Kim et al., 2016). Activated STAT3 induces resistance by promoting EMT, increasing anti-apoptotic signaling, and regulating miRNAs and exosomes (Yin et al., 2017; Wang L. et al., 2018). For example, STAT3 can promote the secretion of exosomes by upregulating Rab, which induces platinum resistance in ovarian tumors (Dorayappan et al., 2018). In addition, activated STAT3 can regulate the delivery of drugs by triggering vascular abnormalities (Nagathihalli et al., 2015). Notch is another crucial adaptive signaling pathway responsible for the TME-induced chemotherapy resistance of tumor cells (Meurette and Mehlen, 2018). Exosomes derived from the stroma deliver Jag1, a Notch ligand, activate the Notch pathway to trigger resistance in breast tumor cells (Boelens et al., 2014). CAFs also can release IL-6 to activate the Notch pathway in breast tumor cells (Studebaker et al., 2008). Induction of Notch3 is also relevant to CSCs transformation in liver cancer (Lin et al., 2017). Accordingly, these signaling pathways response to TME may be potential targets from a therapeutic perspective.

## Targeting the Tumor Microenvironment for Therapy

Multiple preclinical studies implicate the TME as a potential therapeutic target (Jin and Jin, 2020). For instance, multiple strategies of combined therapy related to the TME have shown interesting potential (Table 1). In the TME, tumor cells usually hijack CAFs, ECM, the immune system, hypoxia, and acidosis-related pathways to escape immune surveillance. For example, dysregulated immune signaling pathways have been proven to impair several processes including antigen presentation and T cell infiltration. Thus, targeting the TME might have the potential ability to reverse the resistance of tumor cells.

**TABLE 1:** Combination therapies against cancer used in recent clinical trials.

Conventional drug	Combination therapy	Cancer targeted
Statin	Targeted therapy such as erlotinib, sorafenib, fulvestrant, aromatase inhibitors, anti-HER2	NSCLC, HCC, ESCA, and breast cancer
	Radiotherapy	GBM
	Chemotherapy such as topotecan, zoledronate, bendamustin, and capecitabine	TNBC, pediatric solid and CNS tumor, rectal cancer
Metformin	Targeted therapy such as lanreotide, toremifene, trametinib, gefitinib, and lapatinib	NSCLC, CRC, TNBC, LAML, UCEC and melanoma, kidney cancer, breast cancer
	Radiotherapy	HNSCC, CESC, lung cancer
	Chemotherapy such as docetaxel, oxaliplatin, temozolomide, gemcitabine, and paclitaxel	ESCA, PDAC, GBM, and prostate cancer, breast cancer
Aspirin	Targeted therapy such as osimertinib	NSCLC
	Immunotherapy such as atezolizumab, avelumab, bevacizumab, and ipilimumab	HNSCC, TNBC, and OV
Celecoxib	Targeted therapy such as gefitinib, depsipeptide, toripalimab	ESCA, CRC, HNSCC, NSCLC, OV, and breast cancer
	Radiotherapy	NSCLC
	Chemotherapy such as docetaxel, cisplatin, paclitaxel, and methotrexate	NSCLC, GBM, TNBC, CRC, and PDAC
	Immunotherapy such as nivolumab	NSCLC

### Targeting Cancer-Associated Fibroblasts

Given the critical role of CAFs, the most abundant cell type in the TME, in therapeutic resistance of tumor cells, emerging evidence supports targeting protumorigenic CAF functions as a promising approach for tumor therapy (Truffi et al., 2020). For example, conophylline is used to treat refractory pancreatic cancers by suppressing CAF activity and the proliferation and secretion of cytokines (Zhen et al., 2017; Umezawa et al., 2018; Ishii et al., 2019). The cell surface markers GPR77 and CD10, specifically expressed in CAFs, are involved in chemo-resistance in lung and breast cancer (Vaquero et al., 2018). Treatment with inhibitors of these molecules is a breakthrough in overcoming resistance. For example, using a GPR77-neutralizing antibody to selectively inhibit CAFs is an effective way to restore the sensitivity of drugs. Another strategy is to inhibit the activation of protumorigenic pathways in CAFs. Inhibition of Hedgehog signaling in CAFs successfully enhances the effect of docetaxel chemotherapy in TNBC patients (Cazet et al., 2018). In addition, reversing activated CAFs into a dormant state is also an effective therapeutic strategy. The VDR is considered to be a targeted molecule that regulates the transcriptional process to activate CAFs in pancreatic cancer (Yamashita et al., 2012). Interestingly, compared with gemcitabine alone, the synergistic effect of gemcitabine and a VDR ligand suppresses fibrosis and inflammation, and restores the sensitivity of gemcitabine by increasing tumor uptake, thereby improving the survival rate to 57% (Yamashita et al., 2012; Yang et al., 2016). Studies have also found that nanoparticles loaded with a secreted sTRAIL can reduce the activation of CAFs in pancreatic cancers (Yang et al., 2016). Currently, some clinical trials targeting CAFs are being implemented. The agent RO6874281 is an interleukin-2 variant targeting FAP that is being evaluated for its clinical benefit in combination with atezolizumab, gemcitabine, or vinorelbine in the treatment of advanced tumors. The synergistic treatment of RO6874281 with trastuzumab or cetuximab in patients with head and neck cancer or breast tumors is also in clinical trials.

### Targeting the Extracellular Matrix

Blocking the communication between tumor cells and their environment by targeting adhesion molecules, proteolytic enzymes, and ECM components has been demonstrated as an efficient strategy for tumor therapy (Pickup et al., 2014; Hirata and Sahai, 2017). For example, suppression of the integrin-mediated bidirectional transmitting signals between cells and ECM has been used to prevent therapeutic resistance (Kechagia et al., 2019). Blocking the activity of β1 integrin with monoclonal antibody AIIB2 can significantly increase the outcome of HER2-targeting agents as well as radiotherapy (Park et al., 2008; Weigelt et al., 2010; Hirata and Sahai, 2017). The antagonist of integrin α4β1 and α4β7, natalizumab, has been proven to recover the sensitivity of chemotherapy drugs in malignant tumors, including acute lymphoblastic leukemia (AML) and ovarian tumor (Podar et al., 2011; Hsieh et al., 2013; Scalici et al., 2014; Hirata and Sahai, 2017). Matrix metalloproteinases (MMPs), one of the major ECM components, function as proteases to detach cancer cells out of the ECM, participating in tumor development, metastasis, and resistance (Najafi et al., 2019). Several agents targeting MMPs have been developed for treating advanced carcinomas including incyclinide, JNJ0966 for MMP-9, and the antibody Fab 3369 for MMP-14 (Ling et al., 2017; Scannevin et al., 2017).

TGF-β signaling is another pathway which mediates communication between tumor cells and their ECM. Therefore, anti-TGFβ drugs, including neutralizing antibodies, ligand traps, small-molecule kinase inhibitors, and antisense oligonucleotides (AONs), are a potential strategy for improving therapeutic efficacy. Blocking TGF-β by 1D11, a TGF-β neutralizing antibody, can improve the intra-tumoral penetration of both chemotherapeutic drugs and nanotherapeutic agents by normalizing the tumor interstitial matrix, thereby resulting in a better control of tumor growth (Liu et al., 2012). Galunisertib (LY2157299), an oral small-molecule inhibitor of TβRI, sensitizes colorectal cancer cells to RTK inhibitors (Brunen et al., 2013; Colak and ten Dijke, 2017). Blockade of TGF-β has been found to promote the anti-tumor activity of CD8^+^ T cells, thereby reversing the resistance to a PD-1/PD-L1 blockade in the TME (Chen et al., 2018). Moreover, TGF-β blockade by expressing dominant-negative TGF-β receptor II enhances the efficacy of TCR gene therapy against advanced cancers (Bendle et al., 2013). Both fresolimumab, a pan-TGF-β neutralizing antibody, and LY3022859, an anti-TβRII IgG1 monoclonal antibody, exhibited anti-tumor activity in a phase 1 clinical trial for various cancers (Colak and ten Dijke, 2017). TβR inhibitors, including LY2157299 and PF06952229, have being tested in clinical trials for patients with advanced or drug-resistant cancers (NCT03685591) (Liu et al., 2021).

However, some studies suggest that targeting the ECM has limited outcomes in advanced malignancies such as GBM, melanoma, and prostate tumors, suggesting that different tumors with different extracellular matrices can exhibit different results (Eisele et al., 2014; Stupp et al., 2014; Hirata and Sahai, 2017). Therefore, further investigation and clinical trials are necessary to ensure treatment using ECM-targeting strategies are effective and safe.

### Targeting the Immune System

As mentioned above, the immune system in TME dramatically affects the response of tumors to various treatment approaches. Therefore, multiple strategies based on targeting the immune system have been used to tackle cancers: (i) inhibiting the recruitment of macrophages to tumor tissues; (ii) blocking the differentiation of macrophages toward TAMs; (iii) enhancing the anti-tumor activity of the immune system (Joyce, 2005; Tsai et al., 2014; Roma-Rodrigues et al., 2019). Several studies indicate that combination treatment using conventional therapies and immunotherapy achieves satisfactory clinical outcomes. Indeed, the combination of chemotherapy drugs and immune checkpoint inhibitors (ICIs) has shown a better result compared with chemotherapy alone (Ridker et al., 2017; Tulotta and Ottewell, 2018). Consistently, targeting CTLs with pembrolizumab enhances the clinical efficacy of cisplatin in drug-resistant patients (Ridker et al., 2017). These promising results have prompted a series of clinical studies to validate this combination therapy strategy. The suppression of MDSCs by anti-CSF-1R neutralizing antibodies or small molecule inhibitors has been shown to reduce tumor growth and metastasis (Noy and Pollard, 2014; Szebeni et al., 2016; Ridker et al., 2017). Immune checkpoint molecules in NKs were also reported to be potential targets for immunotherapy (Cao et al., 2020). To date approximately 174 clinical trials involving CTLA-4 and 750 involving PD-1 and its receptor PD-L1 have been reported (as reviewed in Boohaker et al., 2018; Darvin et al., 2018; Chen T. et al., 2019). A combination with blocking antibodies against CTLA-4 and PD-1 results in significantly higher response rates and improved survival in patients with metastatic melanoma (Wolchok et al., 2013; Horvat et al., 2015; Larkin et al., 2015; Robert et al., 2015). Moreover, Kineret, an IL-1 receptor antagonist that is often used for rheumatoid arthritis, has exhibited promising results for breast cancer patients (Tulotta and Ottewell, 2018). The anti-IL-1 antibody canakinumab, which is often used for inflammatory diseases, has shown improved clinical outcomes in lung cancer (Ridker et al., 2017). More recently, canakinumab has been proposed for the treatment of highly aggressive tumors NSCLC and triple negative breast cancer (Houriet et al., 2017; Dhorepatil et al., 2019; Paz-Ares et al., 2019; Schenk et al., 2019; Sehested et al., 2019). In addition, combining traditional therapies with immune therapies has shown potential to reverse resistance (Patel and Minn, 2018; Gomez et al., 2020). For example, the combination of HMTs with immune therapy has shown efficacy for various cancer in pre-clinical studies (Zingg et al., 2017; Patel and Minn, 2018; Gomez et al., 2020). Treatment with EZH2 inhibitor and antibodies blocking CTLA-4 was reported to reverse immunosuppressive effects and significantly improve survival in preclinical models (Zingg et al., 2017). Moreover, DNMTi or HDACi treatment was reported to sensitize resistant cancer cells in mouse models of melanoma and lung adenocarcinoma (Chiappinelli et al., 2016; Strick et al., 2016; Zheng et al., 2016; Stone et al., 2017). The combination of HDACis, DNMTis, anti-CTLA4, and anti-PD1 together showed significantly improved rates of survival, with 75% of mice with tumors being cured (Kim et al., 2014; Zheng et al., 2016). Thus, targeting the immune system, especially in combination with traditional chemotherapy to promote survival and reverse resistance, may prove to be a safe and effective strategy for multiple types of tumors, benefitting more patients.

### Targeting Hypoxia and Acidosis

As mentioned above, low oxygen pressure and acidosis conditions in the TME dramatically affect a tumor’s response to treatment. Therefore, it is rational to manipulate hypoxia and acidosis conditions in the TME to hinder tumor progression (Paolicchi et al., 2016; Roma-Rodrigues et al., 2019). Accordingly, topotecan, an inhibitor of HIF-1α, has been used to cure advanced tumors including ovarian and small cell lung cancers (Roma-Rodrigues et al., 2019). Topotecan has also been used in a clinical study for the treatment of refractory advanced solid neoplasms expressing HIF-1α. Additionally, several clinical trials based on intervention against hypoxia are underway, including the evaluation of everolimus, which downregulates HIF-1α, in combination with lenvatinib in renal cancer (NCT01206764), as well as an evaluation of the metformin outcomes in tissue oxygenation of head and neck cancer (NCT03510390) (Roma-Rodrigues et al., 2019). The acidified environment has been considered to protect tumor cells from chemotherapy by affecting the concentration of drugs (Kolosenko et al., 2017). Hence, recent clinical trials have focused on alteration of the acidification environment by targeting carbonic anhydrases, a family of enzymes that regulate the pH of active cells/tissues in tumors (Supuran, 2018). For example, the synergistic treatment of acetazolamide, a carbonic anhydrase inhibitor, with radiotherapy has been tested in lung cancer (NCT03467360) while the combination of acetazolamide and temozolomide has also been trialed for brain cancer (NCT03011671).

## Discussion

In this review, we have summarized the current insights into how the TME regulates cancer resistance to therapies. The adaptive resistance to cancer treatment driven by the TME may play a vital role in tumor recurrence and metastasis. Therefore, a variety of approaches targeting the TME have been developed to reverse resistance to radiotherapy, chemotherapy, and immunotherapy (Xu et al., 2015; Zhang et al., 2015; Hu et al., 2017; Tan et al., 2018; Bhat et al., 2021). Hijacking the TME to increase drug delivery has also been demonstrated to enhance the efficacy of chemotherapeutic drugs (Fang et al., 2018; Amini et al., 2019). Although a large number of studies have shown the successful application of manipulating the TME to overcome resistance, there are several key questions that still need to be resolved. Firstly, the mechanisms underlying adaptive and non-adaptive resistance need further investigation in a real TME. To date, most of the studies have been based on resistant cells *in vitro*, which excludes the factors in the TME that may be vital for acquired resistance *in vivo*. Co-culture of tumor cells with other types of cells within the TME and tumor-microenvironment-on-a-chip (TMOC) models can only partially mimic the real TME. More studies should be conducted using appropriate mouse models, or the use of human organoids isolated from patient biopsies, to fully understand the role of TME adaptive and non-adaptive resistance (Papapetrou, 2016; Mazzarella and Curigliano, 2018; Vlachogiannis et al., 2018). Secondly, since the TME changes dynamically during the development and treatment of tumors, understanding and monitoring the factors that influence the therapeutic effects of targeting the TME is necessary to improve patient safety and survival outcome. Lastly, some drawbacks of targeting the TME, such as immune-related side-effects, may lead to the cessation of treatment (Liu et al., 2017; García-Aranda and Redondo, 2019). Hopefully, as our understating of intrinsic biology of the TME is improved, we will be more capable of preempting or reversing cancer therapy resistance.

## Author Contributions

NX, JL, and PW conceptualized the manuscript. PW and WG collected the literature and wrote the manuscript. MS and WZ made the figures. EN made significant revisions to the manuscript. All authors read and approved the final manuscript.

## Conflict of Interest

The authors declare that the research was conducted in the absence of any commercial or financial relationships that could be construed as a potential conflict of interest.
